# Positive melioidosis serology in a patient with adult onset Still’s disease: a case report of a diagnostic dilemma

**DOI:** 10.1186/s41927-018-0044-5

**Published:** 2018-12-10

**Authors:** Harsha Anuruddhika Dissanayake, Gayani Premawansa, Enoka Corea, Inoshi Atukorale

**Affiliations:** 1 University Medical Unit, National Hospital of Sri Lanka, Colombo, Sri Lanka; 2 Colombo North Teaching Hospital, Ragama, Sri Lanka; 3 Department of Microbiology, Faculty of Medicine, University of Colombo, Colombo, Sri Lanka; 4 Department of Clinical Medicine, Faculty of Medicine, University of Colombo, Colombo, Sri Lanka

**Keywords:** Adult onset Still’s disease, Melioidosis, False positive antibodies

## Abstract

**Background:**

Autoimmune disorders are known to produce false positives in serological tests for infections. Aetiological association between infections and autoimmunity, increased susceptibility to infectious and autoimmune disorders with immune dysregulation and non-specific polyclonal expansion of B cells with autoimmunity may cause confusion in diagnosis and patient management. We report a patient with Adult Onset Still’s Disease (AOSD) presenting with rising melioidosis antibody titres that caused diagnostic confusion.

**Case presentation:**

A forty-nine-year-old female presented with prolonged fever, sore-throat, large joint arthritis, lymphadenopathy, hepatomegaly and transient rash. She had elevated inflammatory markers and a rising melioidosis antibody titre. The patient responded poorly to prolonged course of appropriate antimicrobials but showed rapid and sustained improvement with glucocorticoids.

**Conclusion:**

Positive melioidosis serology could have been due to a co-infection or false positive antibody reaction due to non-specific B cell expansion or an indicator of true infection that triggered the immune dysregulation to develop AOSD.

## Background

Melioidosis is an emerging infection in the tropics caused by *Burkholderia pseudomallei*. Diagnosis of melioidosis is a challenge [1]. Although isolation of the organism in culture is the gold standard, it has a low sensitivity and negative predictive value [2]. Detection of antibodies against melioidosis in serum provides valuable supportive evidence. However, serology alone is not reliable enough for confirmation. The major disadvantage of serological tests is the false positive results. This is may occur due to the presence of antibodies in people living in endemic areas, cross reactivity with antibodies to different organisms or in association with autoimmune disorders. We report a patient with adult onset Still’s disease (AOSD) who developed antibodies against *B. pseudomallei,* a phenomenon not described previously.

## Case presentation

A forty-nine-year-old female from a suburban community in Sri Lanka presented with insidious high grade intermittent fever with chills and rigors for 2 months. She experienced one to two febrile episodes daily with complete defervescence in between. She also had anorexia, weight loss, sore-throat and symmetrical large joint arthritis without morning stiffness. Small joints and axial skeleton were spared. She also noticed an itchy desquamating erythematous rash over back of the trunk and proximal limbs. Erythematous patches were transient and recurring but did not temporally correspond to febrile peaks. The patient did not have any symptoms referable to a focus of infection and did not report photosensitivity, Raynaud phenomenon, past history of tuberculosis, or high risk sexual behaviours.

The patient was averagely built (BMI: 23.1 kg/m^2^), febrile (39.9 °C), ill and pale. A firm 1.5 cm lymph node in the right posterior cervical group was noted. Throat was non-inflamed. Erythematous macules noted over the trunk and proximal limbs were transient. Symmetric arthritis affected elbow, wrist and knee joints. A smooth non-tender 2 cm hepatomegaly was noted. The rest of the examination was unremarkable.

**Fig. 1:**
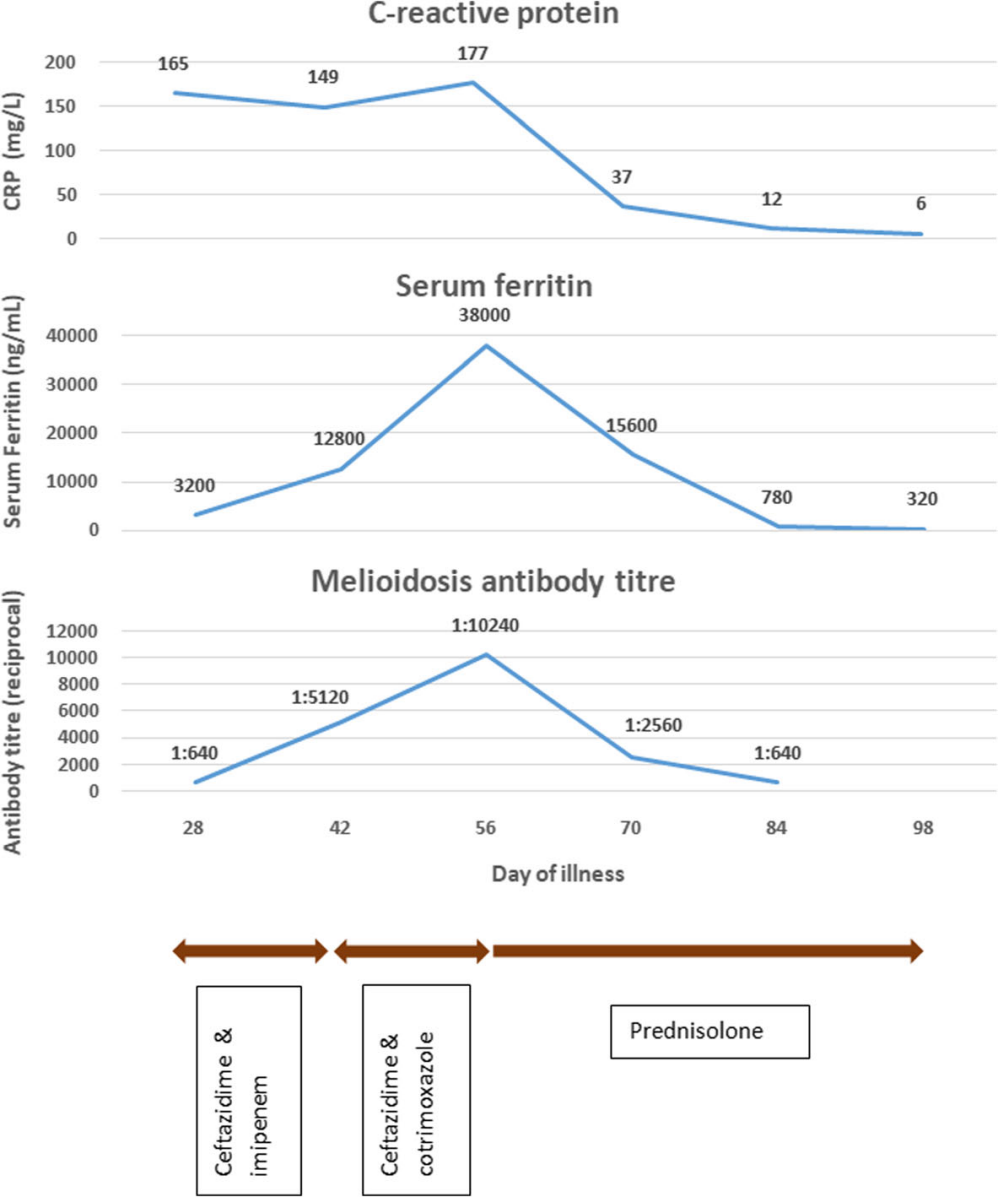
C-reactive protein, ferritin and anti-melioidosis antibody titres, Changes in CRP, ferritin and antibodies against melioidosis over time are illustrated. Treatment instituted are noted at the bottom

Investigations revealed a normocytic normochromic anaemia, neutrophil leukocytosis with toxic changes, reactive thrombocytosis, elevated ESR (110 mm 1st hour), CRP (165 U/L) and ferritin (3200 U/L). Renal function was normal and liver enzymes were mildly elevated (AST 66 U/L, ALT 57 U/L). Auto antibody panel, including rheumatoid factor, antinuclear antibodies (ANA), dsDNA antibodies, pANCA and cANCA were negative. Contrast enhanced computerized tomography of the neck, chest, abdomen and pelvis demonstrated enlarged cervical lymph nodes and fatty liver. Radiographs of large joints were normal. Biopsy of the lymph node showed reactive lymphoid hyperplasia with no evidence of neoplastic changes, suppuration or granuloma. Bone marrow aspiration and trephine showed no abnormalities. Blood and bone marrow cultures for bacteria, tuberculosis, brucellosis, melioidosis and fungi were negative. Serum protein electrophoresis showed polyclonal gamma-globulinaemia and reduced albumin fraction. As she had been exposed to flood water 2 weeks prior to symptom onset (compatible with incubation period of 1–3 weeks) and several cases of melioidosis had been reported in her residential area, antibodies against *Burkholderia pseudomallei* were tested. It turned positive (1:640, indirect haemagglutination) and titre continued to rise over time (Fig. 1).

Febrile illness, possible exposure to infectious agents during floods and elevated inflammatory markers necessitated consideration of empiric antibiotic therapy. The patient was treated with ceftazidime (2 g 6 hourly IV) and imipenem (1 g 8 hourly IV) for 14 days and ceftazidime and cotrimoxazole (1440 mg bid orally) for another 14 days, due to positive serology for melioidosis. However she did not show clinical improvement. Fever persisted and inflammatory markers remained elevated. Serum ferritin and melioidosis antibody titre continued to rise exponentially. However repeated peripheral blood cultures for melioidosis did not isolate any bacteria and repeated imaging did not reveal a focus of infection.

Despite a month of broad spectrum antibiotics she remained febrile with persistently elevated inflammatory markers. In retrospect, AOSD was considered as a possible diagnosis. She showed partial response to NSAIDs, further favouring this diagnosis. Subsequently, she was commenced on high dose steroids (prednisolone 1 mg/kg/day). Within 2 days she achieved complete defervescence and made a good clinical recovery. Serum ferritin level and melioidosis antibody titre declined over time (Fig. 1). After one month of steroid therapy she remained afebrile, but had mild residual large joint arthritis with minimal functional impairment. Methotrexate was commenced and steroids were tapered over next 2 months and at 6 months she remained asymptomatic on methotrexate with normal inflammatory markers. Long term follow up was arranged.

The final diagnosis of AOSD was made based on clinical features and exclusion of other connective tissue disorders, neoplasms and infections. During the course of her illness the patient did not develop Macrophage Activation Syndrome, a serious complication of AOSD.

## Discussion

AOSD is a diagnosis of exclusion. The patient had few atypical features of AOSD, namely, a pruritic rash that did not parallel the febrile episodes and absence of typical twice a day fever spikes. However, the presence of prolonged fever, sore-throat, large joint arthritis, lymphadenopathy, hepatomegaly, elevated inflammatory markers including ferritin, neutrophil leukocytosis, elevated liver enzymes, negative antinuclear antibodies and rheumatoid factor along with exclusion of neoplasms (imaging and histology), other infections (imaging and culture) and autoimmune disorders (negative autoantibody profile) confirmed the diagnosis of AOSD. The systemic score was 4.0 predicting favourable outcome [3].

Isolation of the organism by culture is the gold standard for diagnosing melioidosis. Specificity and positive predictive value of melioidosis antibody testing are modest (68.7–91.3% sensitivity, 17.5–40.9% positive predictive value) and depends on assay methods [4]. False positive results in melioidosis serology have been reported in disease endemic areas [4–6]. However, progressive rise and subsequent decline in antibody titre makes it unlikely to be due to background exposure in our patient.

The mechanism by which the patient produced a serological response against *B pseudomallei* can be three-fold. First, this may have been a false positive result due to antibodies produced by activated polyclonal B cells in AOSD. Second, onset of AOSD would have led to reactivation of latent melioidosis infection. Third, meliodosis infection would have triggered an immune dysregulation and precipitated the onset of AOSD.

Autoimmune disorders are characterized by production of autoantibodies that target self-antigens. It is possible that these antibodies cross react with microbial antigens and produce a false positive result when tested for microbial antibodies. Examples include false positive HIV antibodies in systemic lupus erythematosus (SLE), discoid lupus and mixed connective tissue disease [7], measles antibodies in SLE and chronic active hepatitis [8] and falsely positive fluorescent treponemal antibody absorption in SLE [9, 10].

However, unlike many other autoimmune disorders, AOSD is an auto-inflammatory disease characterized by dysregulation of innate immune system and over expression of chemokines such as IL-1, IL-6 and IL-17 [11]. It is not associated with autoantibody production, although rare cases of AOSD with positive ANA [12] and positive anti-golgi antibodies [13] have been reported. Dysregulated innate system and over-expressed chemokines can activate memory B cells previously sensitized against melioidosis to produce antibodies. IL-6, a key pathogenic mediator in AOSD [11], promotes polyclonal B cell maturation to plasma cells [14] leading to hypergammaglobulinaemia. It is possible that in our patient, plasma cells producing melioidosis antibodies were activated. A report of false positive *Treponema pallidum* haemagglutination following treatment with intravenous immunoglobulin therapy, provides further evidence to the possibility of having specific antibodies in a non-specific hypergammaglobulinaemic state [15].

Alternatively, the patient may have had latent melioidosis infection which reactivated with the onset of AOSD and dysregulation of innate immune system which would have held the latent infection quiescent. Aggressive and prolonged antibiotics would have controlled the infection while fever persisted due to AOSD that remained untreated. Although classical presentation of melioidosis is with pneumonia or multiple abscesses, altered innate system would have blunted the host’s ability to mount a pyogenic immune response and abscess formation.

Finally, primary meliodosis infection in this patient would have triggered an innate immune system dysregulation precipitating the auto-inflammatory response resulting in AOSD. Antibiotic therapy would have treated the infection. In fact possible infectious aetiologies have been proposed in AOSD including several viruses and bacteria including *Mycoplasma pneumonia, Chlamydia pneumonia, Brucella abortus, Borrelia burgdorferi, Bartonella henslae* and *Yersinia enterocolitica* [15]. *B. pseudomallei* has not been described before as a possible aetiology of AOSD. However, other similar gram negative bacteria have potentially been linked to causation of AOSD, a feature *B pseudomallei* may also share. In fact, *Burkholderia* species have been causatively linked to a cutaneous vasculitis [16].

Considering the parallel changes of ferritin and melioidosis antibody titre (Fig. 1), it is unlikely that infection preceded and triggered the onset of AOSD. Reactivation of melioidosis with onset of AOSD also appears less likely given the poor response the antibiotics and prompt response to steroids. False positive rise of melioid antibodies therefore remains the most likely explanation for this observation.

## Conclusion

This case illustrates the possible interactions of infectious and autoimmune disorders in pathogenesis, overlap in clinical manifestations and dilemmas in diagnosis. This also highlights the importance of judicious selection of diagnostic tests in evaluating patients. Serology for melioidosis should be performed in the appropriate clinical context and isolation of the organism by culture should remain the preferred method for confirmation. Serological tests should be used cautiously, considering the confusion caused by false positive results in autoimmune diseases. Further studies are required to explore this phenomenon and to establish the aetiological associations of infections and autoimmune disorders.

## Abbreviations

ALT: Alanine transaminase
ANA: Anti-nuclear antibodies
AOSD: Adult onset Still’s disease
AST: Aspartate transaminase
cANCA: Cytoplasmic anti-neutrophil cytoplasmic antibodies
CRP: C – reactive protein
dsDNA: Double stranded DNA
ESR: Erythrocyte sedimentation rate
HIV: Human immunodeficiency virus
NSAIDs: Non-steroidal anti-inflammtory drugs
pANCA: Perinuclear anti neutrophil cytoplasmic antibodies
